# Examination of the Pelvic Viscera in Virgins

**Published:** 1898-09

**Authors:** William F. Waugh

**Affiliations:** Chicago, Ill.


### EXAMINATION OF THE PELVIC VISCERA IN VIRGINS.

BY WILLIAM F. WAUGH, M.D., CHICAGO, ILL.

One of the physician's most unpleasant duties is that of making a vaginal examination of a young and innocent girl. No matter what may be the need, it is a thing one is always reluctant to do, and is usually put off until it can not be avoided. And herein much harm may be done, for there are many ailments that require prompt relief, and the delay exacted by modesty may entail much avoidable suffering, or even lifelong distress.

One case the writer recollects was that of a girl who was brought to him on account of a leakage of urine. This had been complained of for weeks, but the girl was not brought to his consulting-room until the excoriation rendered her miserable and the odor excluded her from society. The uterus had been retroverted by an overfilled bladder neglected during a day's shopping; the bladder was paralyzed, the urine had found a fistulous exit, the uterus was tightly glued in its abnormal place by exudates. A long course of treatment, some surgery, much pain, distress and mortification, finally resulted in a not very satisfactory cure. Incidentally, the treatment involved the total destruction of the hymen and complete dilatation of the vagina. And all this would have been avoided if the patient had applied for relief the day the uterus was first retroverted.

On the other hand the cases are not unusual where the symptoms are such as to compel the physician to insist on such an examination, to exclude the possibility of local derangements. The result may be to show that no local disease exists, and the shame and bodily injury, in the demolition of proofs of virginity, were unnecessary except to negatively confirm the diagnosis. But the doctor will hardly be held guiltless in such cases if he fails to find conditions that will acquit him for making the examination.

My object in writing this is to suggest that most of the objections to such examinations may be obviated.

First, anesthetize the patient. Let her modesty be spared the shock of conscious unveiling. It is a simple and easy matter to administer a few whiffs of bromic ether, and the short anesthetic period of this agent is yet long enough for the doctor to accomplish this part of his task. There is no special danger about bromic ether, nor are there any dangerous or unpleasant sequels.

Second, let the examination be made through the rectum. There are certainly very few pelvic conditions in which a thorough investigation can not be made with the finger in the rectum and the other hand upon the abdomen, the clothing being properly loosened. In some instances much more may be learned of the uterus and ovaries than by the vaginal route. Besides it may often be that the symptoms in part or altogether depend upon disease of the rectal tissues, or on constipation, that would be otherwise overlooked. Finally, a method that avoids touching the genitals or interfering with the hymen is indubitably preferable.—*The Medical Council.*

